# The Psychosocial Impact of Climate Change and natural disasters

**DOI:** 10.1192/j.eurpsy.2024.979

**Published:** 2024-08-27

**Authors:** D. B. Ghosh Dastidar

**Affiliations:** Psychiatry, Calcutta National Medical College, Kol, India

## Abstract

**Introduction:**

In this study we have studied the impacts of natural disaster yash on the development of PTSD in a rural hamlet of West Bengal.

**Objectives:**

Correlation of natural calamity and trauma ie post traumatic stress disorder in exposed population.

**Methods:**

Setting of the study was a relief camp operated for victims of climate change and natural disasters ie cyclone yash 2021.Tool for data collection - PCL 5 questionnaires, socio demographic pro forma, data was analyzed by using statistical SPSS.

**Results:**

Analysis shows that there is statistical correlation between post traumatic stress disorder and subjects exposed to climate change events such as cyclone Yash.

PCL-5 cut-off score between 31-33 is indicative.

**Conclusions:**

Our study clearly demonstrates the impact of climate change and natural disasters on the development of post traumatic stress disorder in the study group.

**Disclosure of Interest:**

None Declared

